# Altering endoplasmic reticulum stress in a model of blast-induced traumatic brain injury controls cellular fate and ameliorates neuropsychiatric symptoms

**DOI:** 10.3389/fncel.2014.00421

**Published:** 2014-12-10

**Authors:** Aric Flint Logsdon, Ryan Coddington Turner, Brandon Peter Lucke-Wold, Matthew James Robson, Zachary James Naser, Kelly Elizabeth Smith, Rae Reiko Matsumoto, Jason Delwyn Huber, Charles Lee Rosen

**Affiliations:** ^1^Department of Pharmaceutical Sciences, School of Pharmacy, West Virginia UniversityMorgantown, WV, USA; ^2^Center for Neuroscience, Health Sciences Center, West Virginia University, MorgantownWV, USA; ^3^Department of Neurosurgery, School of Medicine, West Virginia University, MorgantownWV, USA; ^4^Department of Pharmacology, School of Medicine, Vanderbilt UniversityNashville, TN, USA; ^5^Dean’s Office, College of Pharmacy, Touro University CaliforniaVallejo, CA, USA

**Keywords:** blast-induced traumatic brain injury, blood-brain barrier, endoplasmic reticulum stress, salubrinal, CHOP, apoptosis, prefrontal cortex

## Abstract

Neuronal injury following blast-induced traumatic brain injury (bTBI) increases the risk for neuropsychiatric disorders, yet the pathophysiology remains poorly understood. Blood-brain-barrier (BBB) disruption, endoplasmic reticulum (ER) stress, and apoptosis have all been implicated in bTBI. Microvessel compromise is a primary effect of bTBI and is postulated to cause subcellular secondary effects such as ER stress. What remains unclear is how these secondary effects progress to personality disorders in humans exposed to head trauma. To investigate this we exposed male rats to a clinically relevant bTBI model we have recently developed. The study examined initial BBB disruption using Evan’s blue (EB), ER stress mechanisms, apoptosis and impulsive-like behavior measured with elevated plus maze (EPM). Large BBB openings were observed immediately following bTBI, and persisted for at least 6 h. Data showed increased mRNA abundance of stress response genes at 3 h, with subsequent increases in the ER stress markers C/EBP homologous protein (CHOP) and growth arrest and DNA damage-inducible protein 34 (GADD34) at 24 h. Caspase-12 and Caspase-3 were both cleaved at 24 h following bTBI. The ER stress inhibitor, salubrinal (SAL), was administered (1 mg/kg i.p.) to investigate its effects on neuronal injury and impulsive-like behavior associated with bTBI. SAL reduced CHOP protein expression, and diminished Caspase-3 cleavage, suggesting apoptosis attenuation. Interestingly, SAL also ameliorated impulsive-like behavior indicative of head trauma. These results suggest SAL plays a role in apoptosis regulation and the pathology of chronic disease. These observations provide evidence that bTBI involves ER stress and that the unfolded protein response (UPR) is a promising molecular target for the attenuation of neuronal injury.

## Introduction

Blast-induced traumatic brain injury (bTBI) has been described as the “hallmark injury” of recent wars in Iraq and Afghanistan (Goldstein et al., 2012). The Defense and Veterans Brain Injury Center estimates that approximately 270,000 blast exposures have occurred over the past decade (Farrell-Carnahan et al., 2013). Many blast exposures cause concussive or sub-concussive brain damage and are associated with the shearing of axons (Rosenfeld and Ford, 2010) and the compromise of brain micro-vessels (Chen et al., 2013a). Often these injuries go undetected in soldiers and civilians due to poor understanding of the underlying mechanisms of blast injury and the diagnostic limitations preventing the detection of pathophysiologic changes in *living patients* (Stern et al., 2011).

Blast exposure can cause blood-brain barrier (BBB) dysfunction (Abdul-Muneer et al., 2013; Chen et al., 2013a) and induce short-term inflammatory cascades that promote intracellular Ca^2+^ accumulation (Arun et al., 2013; Abdul-Muneer et al., 2014). Although bTBI is considered a diffuse injury, a majority of damage from our model is localized to the prefrontal cortex (PFC; Turner et al., 2013), where the brain impacts the skull on the contra coup side of exposure (Zhu et al., 2010, 2013). Ca^2+^ perturbations are known to cause endoplasmic reticulum (ER) stress and trigger the unfolded protein response (UPR; Zhang and Kaufman, 2008; Walter and Ron, 2011). Although the UPR has been reported in a model of controlled cortical impact TBI (Farook et al., 2013), the mechanisms of cellular fate are not yet fully elucidated.

Neuropsychiatric behaviors measured in animal models, such as impulsive-like behaviors, are a strong indicator of damage to the rodent PFC (Bidzan et al., 2012; Johnson et al., 2013). Similar personality disorders are often observed in human bTBI patients as well, providing an important research parallel (Vaishnavi et al., 2009). We propose that our clinically-relevant blast model allows us to investigate the process of ER stress and how this response relates to apoptosis and neuropsychiatric disorders.

A common downstream component of the UPR is the C/EBP homologous protein (CHOP), which becomes upregulated during sustained cellular stress to maintain ER homeostasis (Walter and Ron, 2011). The levels of CHOP dictate whether a cell can effectively repair itself, or proceed to apoptosis by regulating pro- and anti-apoptotic mechanisms (McCullough et al., 2001; Galehdar et al., 2010). Acute phase activation of the protein kinase R-like ER kinase (PERK) UPR pathway, and its downstream component growth arrest and DNA damage-inducible protein 34 (GADD34), helps to maintain CHOP within an ideal range to promote cellular repair (Salminen and Kaarniranta, 2010).

Under sustained ER stress, intracellular Ca^2+^ accumulation can trigger apoptosis through a separate cascade involving calpain-mediated Caspase-12 cleavage (Nakagawa et al., 2000). This mechanism is considered separate from the UPR (Badiola et al., 2011), even though both apoptotic cascades share Caspase-3 cleavage as a final common step in undergoing apoptosis (Szegezdi et al., 2006). Using our model, we are interested in determining the mechanism by which bTBI triggers apoptosis and how this relates to the pathology of chronic disease.

This study investigates acute BBB disruption, ER stress mechanisms, apoptosis and impulsive-like behavior following a single blast injury. It has been proposed that bTBI pathophysiology is partly mediated by alterations in BBB permeability (Chen et al., 2013b), which may induce ER stress and trigger the UPR (Begum et al., 2014). The ER stress modulator, salubrinal (SAL), has been used to investigate downstream components of the PERK pathway (Sokka et al., 2007). Our hypothesis is that SAL manipulation of the PERK pathway would maintain CHOP expression within a protective threshold. Balancing CHOP expression should regulate apoptosis and mitigate impulsive-like behavior indicative of blast injury (Kamnaksh et al., 2011). Therefore, treatment options should consider the UPR mechanism for the detrimental sequelae of neuropsychiatric disorders.

## Materials and methods

### Animals

All procedures involving animals (*N* = 144) were approved by the Institutional Animal Care and Use Committee of West Virginia University and were performed according to the principles of the *Guide for the Care and Use of Laboratory Animals*. This work used young adult male Sprague-Dawley rats acquired from Hilltop Lab Animals (Hilltop Lab Animals, Inc.) and weighed ~300–350 g at the time of blast and sacrifice. Animals were acclimated for 1 week prior to experimental use and were housed under 12 h light/dark conditions with food and water available *ad libitum*.

### Blast overpressure exposure

Prior to blast exposure, animals were anesthetized with 4% isoflurane (Halocarbon). The blast was delivered to the right side of the head with the animal’s body oriented perpendicular to the blast tube, and with the peripheral organs protected by a polyvinyl chloride pipe shield. The animals were exposed to a mild blast (0.005” membrane; ~15 psi on incident recordings; ~50 psi on reflected recordings), which was determined, in previous work, to produce microscopic neuronal injury to the contra coup (*left*) side of the brain, with no signs of hemorrhagic injury under gross examination (Turner et al., 2013). Immediately following blast exposure, animals were returned to a holding cage equipped with a homeothermic heating blanket equipped with a rectal thermometer to maintain body temperature at 37°C. Once basic reflexes were restored, animals were returned to the home cage.

### Salubrinal administration

A stock solution of SAL (Tocris) was made in 0.5% dimethyl sulfoxide (DMSO). Such a low concentration of DMSO was chosen to avoid neurological effects (Methippara et al., 2012). SAL was diluted to 100 μM to effectively manipulate the UPR (Boyce et al., 2005). A DMSO concentration of 0.5% was administered to control and bTBI rats 30 min prior to anesthesia. SAL was aliquoted for each intraperitoneal injection at a dose of 1 mg/kg (Sokka et al., 2007; Liu et al., 2014). SAL was administered 30 min prior to anesthesia (SAL), or anesthesia followed by blast exposure (SAL+bTBI).

### Blood-brain barrier permeability assessment

Animals in the BBB assessment group (*N* = 16) were evaluated at three time points following blast exposure: 0.5, 6, 24 h, and control (*n* = 4). Following blast exposure, BBB permeability was assessed using Evan’s Blue (EB; Sigma). Evan’s Blue binds to albumin and is a marker used to detect BBB permeability (Yen et al., 2013). We had previously shown changes in BBB permeability and tight junction protein expression with our model (Lucke-Wold et al., 2014). Animals were anesthetized with 4% isoflurane and maintained with 2% isoflurane throughout the procedure. Saline containing EB (2%, 5 ml/kg) was administered intravenously (femoral vein) 30 min before perfusion. The rats were then transcardially perfused with 0.9% saline for 15 min and brains excised for raw imaging. The PFC was then dissected out and separated by hemisphere. The samples were weighed and homogenized in 0.5 ml of 50% trichloroacetic acid (Sigma). The samples were then incubated for 24 h at 37°C before being centrifuged at 10,000× g for 10 min at 4°C. The supernatant was measured by absorbance spectroscopy at 620 nm. Calculations were based on an external standard reading and extravasated dye was expressed as ng EB/mg brain tissue.

### Quantitative real-time polymerase chain reaction

Rats (*N* = 48) for the gene analysis group were randomly divided into one of two groups: time course (*n* = 24) and control (*n* = 24). The time course group consisted of six time-points post-bTBI: 1.5, 3, 6, 12, 24, and 72 h (*n* = 4). The control group (anesthetized only) used rats for all six time points from the time course study (*n* = 4). All animals were anesthetized with 4% isoflurane and euthanized via decapitation in protease/phosphatase cocktail (Plattner et al., 2006). Brains were rapidly removed with the PFC dissected out, separated by hemisphere and flash frozen in liquid nitrogen. Total RNA was isolated in TRI-Reagent (Sigma) and was tested for quantity and quality using a Nanodrop 2000c spectrophotometer (Thermo Scientific). Reverse transcription was conducted using a high capacity reverse transcription kit (Applied Biosystems). Real-time PCR analyses were performed using a 7500 Real-Time PCR system (Applied Biosystems) in combination with TaqMan® chemistry using the following oligonucleotide primer sets: activating transcription factor 4 (*atf4*) (Rn00824644_g1), CHOP (*ddit3*) (Rn00492098_g1), GADD34 (*ppp1R15A*) (Rn00591894_m1), glial fibrillary acidic protein (*gfap*) (Rn00566603_m1) with 18 s rRNA (Hs99999901_s1) used as an endogenous control (Applied Biosystems). Changes in gene expression were determined using the (ΔΔCt) method with a threshold cycle value of 0.2 normalized to 18 s rRNA.

### Western blotting

Rats (*N* = 20) for the protein analysis group were randomly divided into four different experimental groups: (1) control; (2) SAL; (3) bTBI 24 h; and (4) SAL+bTBI 24 h (*n* = 5). Animals were euthanized and had tissue prepared as previously described. Protein samples were prepared by sonication in hot (85–95°C) 1% sodium docecyl sulfate (Sigma) as previously described (O’Callaghan and Sriram, 2004). The protein concentration of each sample was measured using a bicinchoninic acid (BCA) protein assay kit (Pierce). Samples were run using 30–50 μg of protein/well, depending on the primary antibody, using pre-cast Bolt® Bis-Tris Plus gels (Life Technologies) in combination with 2 X Lammeli sample buffer. Gels were run using the Bolt® Mini tank system (Life Technologies) and transferred to polyvinylidene fluoride membranes (Bio-Rad) using wet electrophoretic transfer cells (Bio-Rad). Membranes were incubated with the following primary antibodies all raised in rabbit: CHOP 1:1000 (Cell Signaling), Caspase-12 1:1000 (Cell Signaling), Caspase-3 1:750 (Cell Signaling), and GADD34 1:1000 (Pierce) overnight at 4°C. Anti-rabbit IgG horseradish peroxidase (HRP)-linked antibody (Cell Signaling) was used as a secondary antibody at a concentration of 1:2000 with gentle shaking for 2 h at room temperature. The rabbit monoclonal antibody β-actin (Cell Signaling) was used as an endogenous control for all samples at a concentration of 1:10,000. Molecular weight determination was conducted using the SeeBlue® Plus2 Pre-stained Standard (Life Technologies). Imaging was conducted using LumiGLO chemiluminescent substrate (Cell Signaling) according to manufacturer’s instructions. Images were converted to 8-bit and analyzed using densitometry with background subtraction and normalized to β-actin using ImageJ software (NIH).

### Immunohistochemistry preparation

Rats (*N* = 12) used for the immunohistochemistry (IHC) group were randomly divided into three experimental groups: (1) control; (2) bTBI 24 h; and (3) SAL+bTBI 24 h (*n* = 4). Histological samples were prepared as previously described (Lucke-Wold et al., 2014). Briefly, the animals were anesthetized by inhalation of 4% isoflurane and maintained with 2% isoflurane throughout the procedure. Animals were then transcardially perfused with 0.9% ice-cold saline for 5 min followed by 10% formalin for 15 min. The brains were subsequently removed and placed in 10% formalin solution for 24 h. Following fixation, the PFC was sectioned on a brain block at 4 mm increments. Sections were then processed with a Tissue Tek VIP 5 automatic processor (Sakura Finetek), and embedded with Tissue Tek TEC (Sakura Finetek) as previously described (Turner et al., 2012). Slices (10 μm) were prepared with the Leica RM2235 microtome (Leica Microsystems), mounted onto slides, and heat fixed for fluorescent staining. A total of 46 coronal sections were prepared per animal.

#### Immunohistochemistry staining

Paraffin was dissolved from slides with 5 min washes in xylene, 100% EtOH, and 95% EtOH followed by 5 min rehydration in dH_2_O. The slides were then quenched with 10% methanol and 10% H_2_O_2_ in Dulbecco’s phosphate buffered saline (DPBS) for 15 min. After quenching, slides were rinsed three times in DPBS for 5 min each. The slides were then placed in permeabilizing solution (1.8% L-lysine, 4% horse serum, and 0.2% Triton X-100 in DPBS) for 30 min. Slides were allowed to dry and the brain slices were circumscribed. Tissue was incubated with primary antibody in DPBS with 4% horse serum overnight. Tissues were washed three times in DPBS and incubated in secondary fluorescent antibody for 3 h. Tissues were then rinsed three times in DPBS and dried overnight. Vectashield mounting media was used to fix the coverslip (Vector Labs). When staining for co-localization, a second set of primary and secondary antibodies were used prior to fixing the coverslip. Primary antibodies were GFAP rabbit mAB (Dako) 1:500, CHOP mouse mAB (Cell Signaling) 1:1600, Caspase-3 rabbit polyAB (Abcam) 1:1000, Caspase-12 mouse polyAB (Cell Signaling) 1:1000, and microtubule associated protein 2 (MAP2) rabbit mAB (Millipore) 1:1000. Secondary antibodies were diluted 1:100 in DPBS and included Alexa Fluor 488 goat anti-rabbit (Life Technologies), Alexa Fluor 594 goat anti-rabbit (Life Technologies), Alexa Fluor 488 goat anti-mouse (Life Technologies), and Alexa 594 goat anti-mouse (Life Technologies). Imaging was performed with a Zeiss Axio Imager 2 (Carl Zeiss Microscopy).

#### Immunohistochemistry quantification

Corrected total cell fluorescence (CTCF) was calculated using ImageJ software (NIH). Briefly, 12 randomly selected areas of the left PFC were outlined and measured, with fluorescent density compared to background readings. Slides from each region were randomly selected by an observer blinded to the experimental groups. The density was adjusted per mean area to give CTCF (Lucke-Wold et al., 2014). For co-localization quantification, the ImageJ plugin titled *Just Another Co-localization plugin* was utilized to calculate a Pearson’s coefficient as well as an overlap coefficient for each sample (Beerten et al., 2012).

### Terminal deoxynucleotidyl transferase-mediated dUTP nick end labeling

Slides for staining were prepared, sectioned and mounted as previously described. We used an apoptosis detection kit to assess the left PFC at 24 h following blast exposure. Staining for apoptosis was completed using the TACS 2 TdT-Dab *in situ* Apoptosis Detection kit (Trevigen) according to manufacturer’s instructions. In brief, paraffin embedded slices were deparaffinized and rehydrated with 5 min incubations in xylenes, 100% ethanol, 95% ethanol, and deionized water each. The slices were then immersed in phosphate buffered saline for 10 min followed by treatments with Proteinase K, quenching solution, labeling buffer, and labeling reaction mixture. The sample was then covered in Strep-HRP solution for 10 min, washed, and then immersed in Diaminobenzidine (DAB) solution for 7 min. The slices were counterstained with 1% methyl green and dehydrated with 10 dips in deionized water, 95% ethanol, 100% ethanol, and xylenes. The slides were coverslipped using Permount™ (Sigma) mounting media and glass coverslips. For quantification of 3,3′-Diaminobenzidine (DAB) staining, regions of the PFC were randomly selected for rats from the different treatment groups. An observer blinded to experimental group, randomly selected 100 total cells. The number of positive cells was reported as a fraction of total cells counted.

### Elevated plus maze

Impulsive-like behavior can be measured with increased exploratory behavior in a rodent model of anxiety (Mosienko et al., 2012). Four groups of rats (*N* = 48) were subject to behavioral analysis: control, SAL, bTBI 7 d, and SAL+bTBI 7 d (*n* = 12). The elevated plus maze (EPM) was set at a height of 60 cm from the floor. The two open arms intersected perpendicular to the two closed arms. Each arm was 50 cm × 10 cm. The closed arms were encased by black siding 30 cm tall. Each rat was placed in the middle of the EPM facing an open arm and tracking was performed for 5 min with AnyMaze™ software (Version 4.7, Stoelting), which pinpointed the location of the animal’s head and body continuously throughout the testing trial. The percent time spent in the open arms, speed, closed arm entries and movement were all recorded and quantified. Increased percent time spent in the open arms was considered a sign of impulsive-like behavior, as previously described (Mosienko et al., 2012; Johnson et al., 2013).

#### Data analysis

Data were analyzed using GraphPad Prism 5.0 (GraphPad Software, Inc.). All data points are shown as mean ± s.e.m. Statistical differences between control and experimental groups were determined by using ANOVA with a Dunnett’s, Tukey’s or Bonferroni’s *post hoc* tests. A two-tailed Student’s *t*-test was used when comparing two conditions only. For DAB staining, a chi-square analysis was used to compare between groups. A power analysis was conducted for all experiments with an α of 0.05 and a β of 0.2 (DSS Research Power Analysis). Sample sizes were determined by the sample effect with behavioral data being set at 0.4 and all other data being set at 0.3. A value of *p* < 0.05 was considered statistically significant for all data analyzed.

## Results

**Figure 1:**
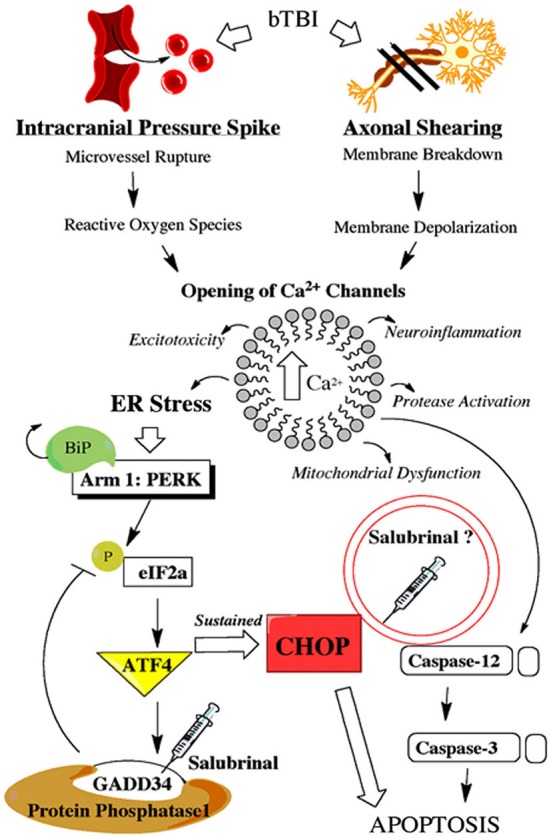
**Schematic shows primary and secondary effects of blast injury**. Blast-induced traumatic brain injury (bTBI) can burst brain microvessels and shear axons. Both primary effects lead to increased intracellular calcium levels which triggers a variety of secondary effects including endoplasmic reticulum (ER) stress. Endoplasmic reticulum stress activates the unfolded protein response (UPR) which consists of three separate adaptive arms that play a time-dependent role in maintaining cellular homeostasis. Following neural injury the protein kinase-like ER kinase (PERK)-mediated UPR is considered the acute phase adaptive arm. This mechanism, along with a link to ER-mediated apoptosis, is displayed with the proposed effects of salubrinal (SAL) included.

The physical force of bTBI can shear axons (Raghupathi and Margulies, 2002), and rupture micro-vessels (Arun et al., 2013). These primary effects can cause neurons to rapidly depolarize and activate voltage gated Ca^2+^ channels, thereby increasing intracellular Ca^2+^ (Gurkoff et al., 2013). Studies using other models of neurotrauma have shown marked BBB dysfunction (Abdul-Muneer et al., 2014), ER stress activation (Farook et al., 2013), and apoptosis (Sabirzhanov et al., 2014). SAL is known to prevent the dephosphorylation of eukaryotic initiation factor 2 alpha (eIF2α; Boyce et al., 2005); however, this agent may directly affect other mechanisms of ER stress (Zhang et al., 2014) or apoptosis (Kessel, 2006). Figure 1 portrays how bTBI may trigger the UPR, as well as the proposed effects of SAL.

### Blast exposure imparts apparent contra coup BBB disruption

The suggestion has been made previously that BBB disruption, or loss of micro-vessel endothelium integrity, may be an inciting event for the molecular changes frequently induced following neurotrauma (Abdul-Muneer et al., 2013; Arun et al., 2013). This may be particularly relevant in models of blast injury in which a pressure wave, and associated surge in vascular flow to the brain, may induce microvascular changes manifested as BBB disruption (Sosa et al., 2013). Following BBB disruption, EB binds to albumin and diffuses into the brain in a location-specific manner consistent with disruption (Yang et al., 2013). Recently, we have shown changes in BBB permeability and tight junction protein expression using our blast model (Lucke-Wold et al., 2014). To further address these issues, we used EB extravasation to assess the brain vasculature of the PFC following blast exposure.

**Figure 2:**
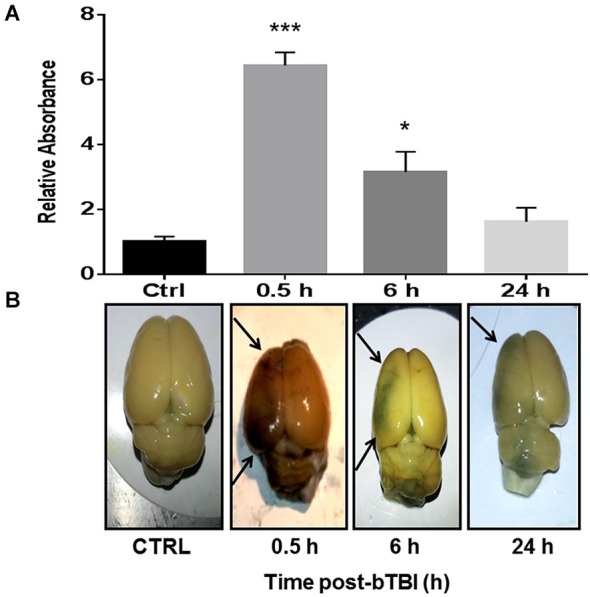
**Blood-brain barrier (BBB) disruption observed in the contra coup brain after bTBI. (A)** Blood-Brain Barrier disruption as evidenced by increased Evan’s Blue (EB) absorbance in the left prefrontal cortex (PFC) at 0.5 h post-bTBI (***p < 0.001 vs. Ctrl) and 6 h post-bTBI (*p < 0.01 vs. Ctrl) (values represent mean ± s.e.m.) (*n* = 4). **(B)** Raw images of EB bound albumin in the left brain parenchyma at acute time points post-bTBI.

One-way ANOVA revealed a significant difference in EB absorbance in the left PFC following blast exposure (*F*_(3,12)_ = 31.350, *p* < 0.001). Dunnett’s *post hoc* analyses revealed bTBI significantly increased EB absorbance in the left PFC at 0.5 h (*q* = 8.844, *p* < 0.001) and 6 h (*q* = 3.491, *p* < 0.05; Figure 2A). Raw images of extracted brains at varying time points (0.5, 6, and 24 h) demonstrate BBB disruption, particularly prominent in the left PFC (Figure 2B). The images provide vivid proof of a contra coup style of injury following bTBI. Because our model’s blast exposure produced no signs of hemorrhagic transformation under gross examination at this severity (Turner et al., 2013), we are certain that these findings indicate a loss in BBB integrity on a microscopic scale.

### Blast upregulates stress response genes atf4, ddit3, ppp1R15A and gfap

**Figure 3:**
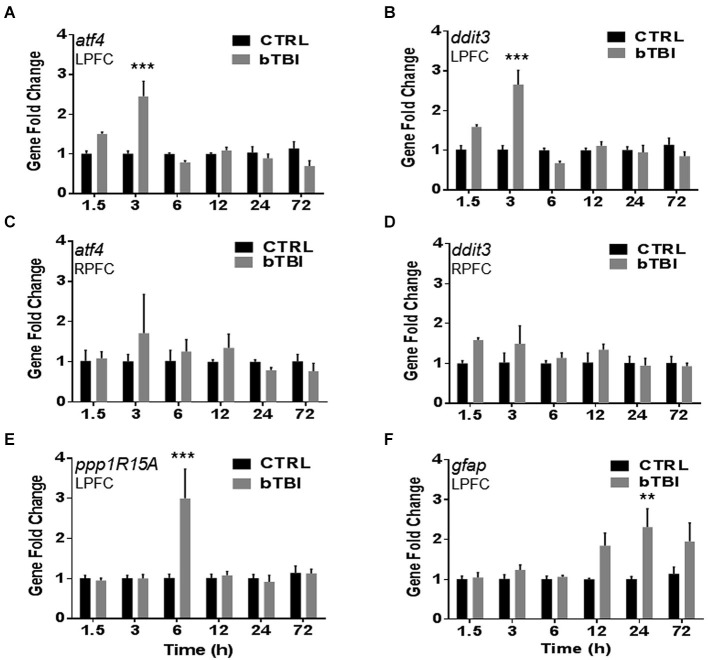
**Blast exposure upregulates stress response genes *atf4*, *ddit3*, *ppp1R15A* and *gfap***. Real-time quantitative PCR time course showed a significant increase in mRNA abundance in the left PFC post-bTBI of stress response genes **(A)** *atf4* (activating transcription factor 4) (ATF4) at 3 h (***p < 0.001 vs. Ctrl at 3 h) and **(B)** *ddit3* (CHOP) at 3 h (***p < 0.001 vs. Ctrl at 3 h). No real differences were observed in mRNA abundance in the right PFC for **(C)** *atf4* (*p* > 0.05) and **(D)** *ddit3* (*p* > 0.05). A significant increase in mRNA abundance was also observed in the left PFC for UPR gene **(E)** *ppp1R15A* (GADD34) at 6 h (***p < 0.001 vs. Ctrl at 6 h), and astrocyte activation marker **(F)** *gfap* (GFAP) at 24 h (**p < 0.01 vs. Ctrl at 24 h) (values represent mean ± s.e.m.) (*n* = 4).

To investigate gene changes following blast exposure, we performed quantitative real-time PCR. A time course was employed to measure acute and sub-acute changes in mRNA abundance of four stress response genes (*atf4, ddit3, ppp1R15A* and *gfap*). *Atf4* encodes for ATF4, *ddit3* encodes for CHOP, *ppp1R15A* encodes for GADD34, and *gfap* encodes for GFAP. Two-Way ANOVA revealed significant differences in left PFC *atf4* mRNA abundance between treatment, time, and interaction (*p* < 0.05). Blast exposure significantly increased the mRNA abundance of *atf4* in the left PFC at 3 h (*t* = 7.694, *p* < 0.001; Bonferroni’s *post hoc* analysis; Figure 3A). Two-Way ANOVA also revealed significant differences in left PFC *ddit3* mRNA abundance between treatment, time, and interaction (*p* < 0.05). Bonferroni’s *post hoc* analysis revealed a significant increase in left PFC *ddit3* mRNA abundance at 3 h post-bTBI (*t* = 7.989, *p* < 0.001; Figure 3B). To further validate our blast model induces a contra coup form of injury we also measured mRNA abundance of *atf4* and *ddit3* in the right PFC. Indeed no differences were observed in the right PFC for *atf4* (Figure 3C), or *ddit3* (Figure 3D). Left PFC mRNA abundance for both *atf4* and *ddit3* quickly returned to baseline by 6 h and remained at this level through 72 h, implying an acute phase stress response (Figures 3A,B).

Two-Way ANOVA revealed significant differences in the left PFC *ppp1R15A* mRNA abundance between time and interaction (*p* < 0.05). Blast exposure significantly increased the mRNA abundance of *ppp1R15A* in the left PFC at 6 h (*t* = 6.022, *p* < 0.001; Bonferroni’s *post hoc* analysis; Figure 3E). Two-Way ANOVA also revealed significant differences in left PFC *gfap* mRNA abundance between treatment and time (*p* < 0.05). Bonferroni’s *post hoc* analysis revealed a significant increase in left PFC* gfap* mRNA abundance at 24 h post-bTBI (*t* = 4.081, *p* < 0.01; Figure 3F). Results indicate blast exposure imparts downstream upregulation of UPR marker, GADD34, with trailing astrocyte activation at a later time point. Astrocyte activity is indicative of neuroinflammation and early stages of cell death.

### SAL attenuates ER stress markers in the contra coup brain after blast

**Figure 4:**
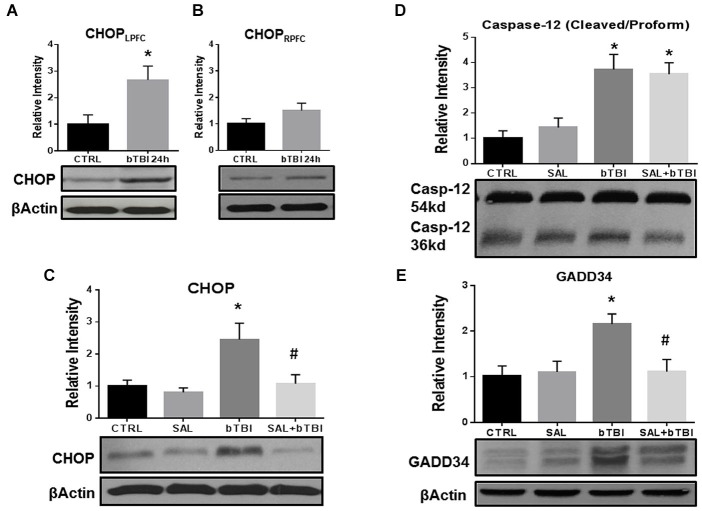
**Salubrinal attenuates ER stress markers in the contra coup PFC after bTBI. (A)** Immunoblots show a significant increase in CHOP expression at 24 h post-bTBI in the left PFC (*p < 0.05 vs. Ctrl). **(B)** No significant differences were observed in the right PFC (*p* > 0.05 vs. Ctrl). **(C)** CHOP expression was significantly increased at 24 h post-bTBI compared to SAL administered alone (*p < 0.05 vs. SAL). Salubrinal administration prior to blast exposure attenuated CHOP expression (#*p* < 0.05 vs. bTBI).** (D)** A significant increase in Caspase-12 cleavage was observed at 24 h in bTBI rats (*p < 0.05 vs. SAL), as well as in SAL+bTBI rats at 24 h (*p < 0.05 vs. SAL). **(E)** GADD34 protein expression was significantly increased at 24 h post-bTBI (*p < 0.05 vs. SAL). Salubrinal administration prior to blast exposure attenuated GADD34 protein expression (#*p* < 0.05 vs. bTBI) (values represent mean ± s.e.m.; normalized to β-actin) (*n* = 4–5).

SAL is a research tool known to inhibit the UPR *in vitro* (Boyce et al., 2005) and *in vivo* (Sokka et al., 2007). In particular, SAL prevents the dephosphorylation of eIF2α by the GADD34 phosphatase complex formation (Hetz et al., 2013). We used this tool prior to blast exposure to alter ER stress and to examine any effects modulating the UPR. Western blot was used to compare CHOP expression in the left and right PFC to further support contra coup injury indicative of bTBI (Zhu et al., 2010, 2013). A significant increase in CHOP expression was measured in the left PFC at 24 h post-bTBI (*t* = 2.625, *p* < 0.05; Figure 4A), but not in the right PFC (*t* = 1.487, *p* > 0.05; Figure 4B; Two-Tailed Student’s *t* Test). A significant difference in CHOP expression was observed with a One-Way ANOVA (*F*_(3,12)_ = 5.775, *p* < 0.01). At 24 h, a significant increase in CHOP expression was seen in the left PFC of bTBI rats (*t* = 3.705, *p* < 0.05), but was attenuated in the left PFC of SAL+bTBI rats (*t* = 3.105, *p* < 0.05; Bonferroni’s *post hoc* analysis; Figure 4C). These findings indicate SAL given alone does not alter the constitutively active form of CHOP; however, stress-activated CHOP can be attenuated when SAL is administered prior to blast exposure.

Endoplasmic reticulum stress markers Caspase-12 and GADD34 were investigated to determine if SAL had any effects on cellular fate following blast exposure. One-Way ANOVA revealed a significant difference in the proteolytic processing of Caspase-12 (*F*_(3,16)_ = 10.230, *p* < 0.001) at 24 h post-bTBI. Caspase-12 cleavage significantly increased in the left PFC of bTBI rats (*t* = 3.696, *p* < 0.05), as well as in the left PFC of SAL+bTBI rats (*t* = 3.393, *p* < 0.05; Bonferroni’s *post hoc* analysis; Figure 4D). A One-Way ANOVA revealed a significant difference in GADD34 protein expression (*F*_(3,16)_ = 5.216, *p* < 0.05) following blast exposure. GADD34 protein expression significantly increased in the left PFC of bTBI rats at 24 h (*t* = 3.136, *p* < 0.05). This effect was mitigated when SAL was administered prior to blast exposure (*t* = 3.107, *p* < 0.05; Bonferroni’s *post hoc* analysis; Figure 4E). Findings suggest SAL may control cellular fate through the modulation of ER stress in a bTBI model.

### SAL modulates fluorescence of apoptosis markers in neurons after blast

CHOP, Caspase-12 and Caspase-3 are all markers of ER-mediated apoptosis (Nakagawa and Yuan, 2000; Nakagawa et al., 2000; Tabas and Ron, 2011). Caspase-12 is initially cleaved by ER stress through a perturbation in Ca^2+^ homeostasis; thereby, activating Caspase-3 and triggering apoptosis (Bernales et al., 2012). To further confirm that bTBI increases CHOP expression, we conducted IHC fluorescent staining in the left PFC and measured CTCF. We also measured CTCF for Caspase-12 and Caspase-3 in the left PFC.

**Figure 5:**
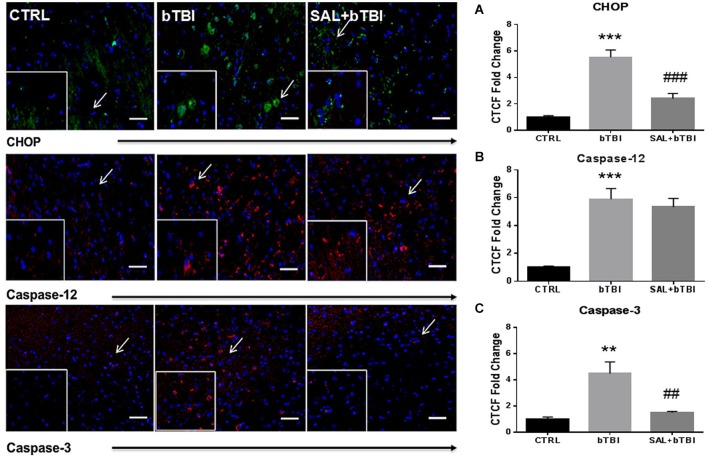
**Salubrinal modulates fluorescence of apoptosis markers after bTBI**. Column of panels display immunofluorescence of apoptosis markers: CHOP (green), Caspase-12 (red) and Caspase-3 (red) (*top* to *bottom*). Row of Panels are separated by experimental group: CTRL, bTBI and SAL+bTBI (*left* to *right*). All images are from the left PFC region and display the blue nuclear counterstain 4’,6-diamidino-2-phenylindole (DAPI). Images are displayed at 20x; arrows demarcate insets at 40x. (Scale bars = 50 μm). **(A)** Immunohistochemistry (IHC) shows bTBI augmented CHOP fluorescence in the left PFC at 24 h (***p < 0.001 vs. Ctrl). Salubrinal administration prior to bTBI attenuated CHOP fluorescence at 24 h (###*p* < 0.001 vs. bTBI). **(B)** Immunohistochemistry shows bTBI increased Caspase-12 fluorescence in the left PFC at 24 h (***p < 0.001 vs. Ctrl). Salubrinal administration prior to bTBI had no significant effect on Caspase-12 fluorescence (*p* > 0.05 vs. bTBI). **(C)** Immunohistochemistry also shows bTBI augmented Caspase-3 fluorescence in the left PFC at 24 h (**p < 0.01 vs. Ctrl). Salubrinal administration prior to bTBI mitigated Caspase-3 fluorescence at 24 h (##*p* < 0.01 vs. bTBI) (values represent mean ± s.e.m.) (*n* = 4; 12 randomly selected areas from left PFC).

Consistent with Western blot findings, IHC analyses showed a significant difference in CTCF for CHOP after blast exposure (One-Way ANOVA; *F*_(2,12)_ = 34.390, *p* < 0.001). Bonferroni’s *post hoc* analyses revealed bTBI significantly increased CHOP CTCF (*t* = 8.099, *p* < 0.001), while SAL pre-administration reduced CHOP CTCF (*t* = 5.592, *p* < 0.001) in the left PFC at 24 h (Figure 5A). Immunohistochemistry analyses also showed that bTBI portrays a significant difference in CTCF for Caspase-12 (One-Way ANOVA; *F*_(2,12)_ = 22.03, *p* < 0.001), and Caspase-3 (One-Way ANOVA; *F*_(2,12)_ = 12.490, *p* < 0.01). Bonferroni’s *post hoc* analyses revealed a significant increase in CTCF for Caspase-12 at 24 h in the left PFC of bTBI rats (*t* = 6.066, *p* < 0.001), as well as SAL+bTBI rats (*t* = 5.366, *p* < 0.001; Figure 5B). SAL also attenuated (*t* = 3.967, *p* < 0.01) the increased Caspase-3 CTCF (*t* = 4.615, *p* < 0.01) at 24 h post-bTBI in the left PFC (Figure 5C). These findings suggest SAL plays an important role in the control of cellular fate through CHOP- and Caspase-3-dependent mechanisms following blast exposure.

**Figure 6:**
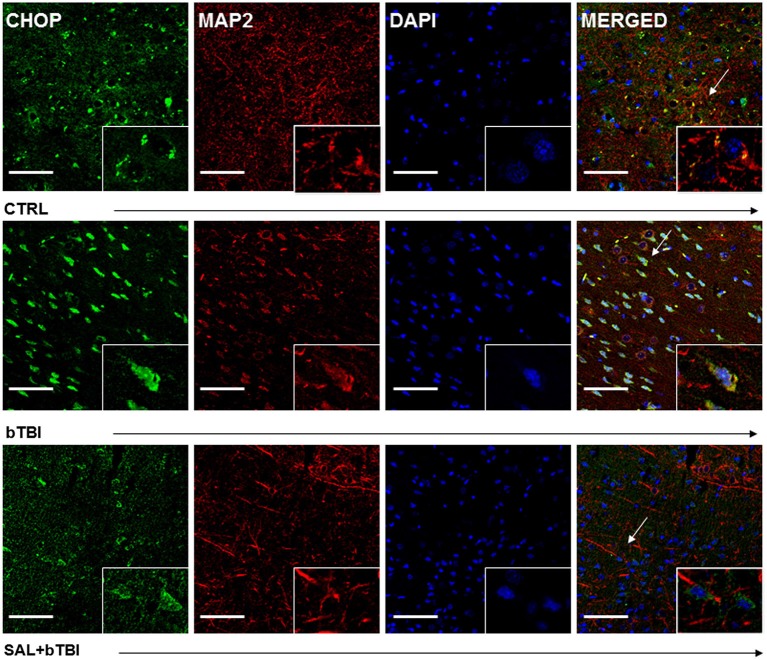
**Salubrinal reduces CHOP and MAP2 colocalization after blast exposure**. Column of panels display immunofluorescence of UPR marker CHOP (green), neuron-specific marker MAP2 (red), nuclear counterstain DAPI (blue), and those three markers merged (yellow) (*left* to *right*). Row of panels are separated by experimental group: CTRL, bTBI and SAL+bTBI (*top* to *bottom*). All images are from the left PFC region with colocalization determined by levels of yellow in merged images. Images are displayed at 20x; arrows demarcate insets at 40x. (Scale bar*s* = 50 μm).

We employed IHC colocalization to determine cell-specific UPR activation. CHOP displayed a moderate colocalization with the neuron-specific MAP2 in the left PFC at 24 h post-bTBI (Pearson’s coefficient, *r* = 0.536; Figure 6). CHOP displayed a weak colocalization with MAP2 in the control (Pearson’s coefficient, *r* = 0.371) and SAL+bTBI groups (Pearson’s coefficient, *r* = 0.233; Figure 6). The results suggest blast exposure increases CHOP protein expression in neurons of the left PFC at 24 h, and also supports the protein and gene data shown previously for CHOP.

**Figure 7:**
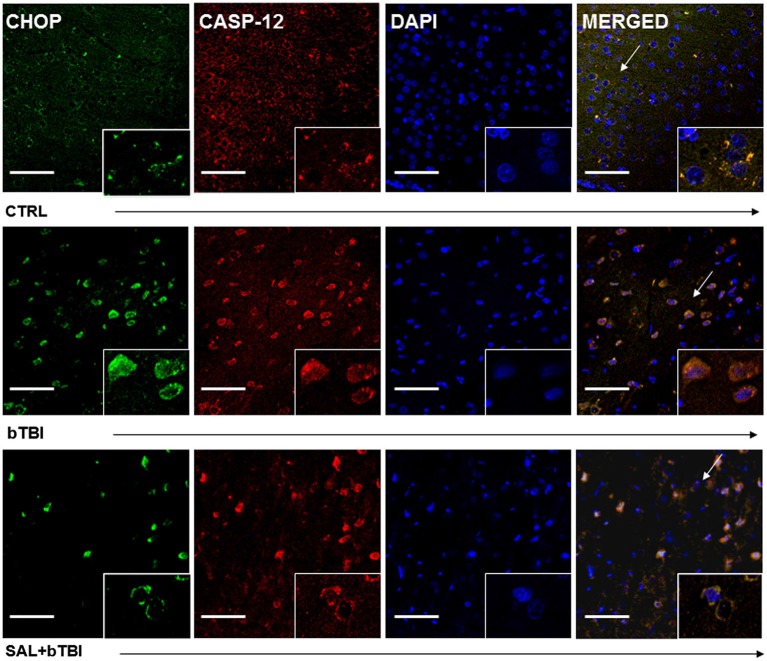
**CHOP and Caspase-12 colocalize regardless of SAL administration prior to bTBI**. Column of panels display immunofluorescence of UPR marker CHOP (green), Caspase-12 (red), nuclear counterstain DAPI (blue), and those three markers merged (yellow) (*left* to *right*). Row of panels are separated by experimental group: CTRL, bTBI and SAL+bTBI (*top* to *bottom*). All images are from the left PFC region with colocalization determined by levels of yellow in merged images. Images are displayed at 20x; arrows demarcate insets at 40x. (Scale bars = 50 μm).

Immunohistochemistry colocalization was also used to determine if CHOP and Caspase-12 activation occur in the same cells. We observed a very weak colocalization between CHOP and Caspase-12 in the left PFC of control rats (Pearson’s coefficient, *r* = 0.177). We discovered a moderate colocalization between CHOP and Caspase-12 at 24 h in the left PFC of bTBI rats (Pearson’s coefficient, *r* = 0.537; Figure 7), as well as a moderate colocalization in the left PFC of SAL+bTBI rats (Pearson’s coefficient, *r* = 0.677; Figure 7). These results suggest CHOP and Caspase-12 to be active within the same cell after blast exposure. Colocalization strengths also suggest SAL may not have a direct effect on Caspase-12 cleavage.

### SAL mitigates caspase-3 cleavage and decreases apoptosis

**Figure 8:**
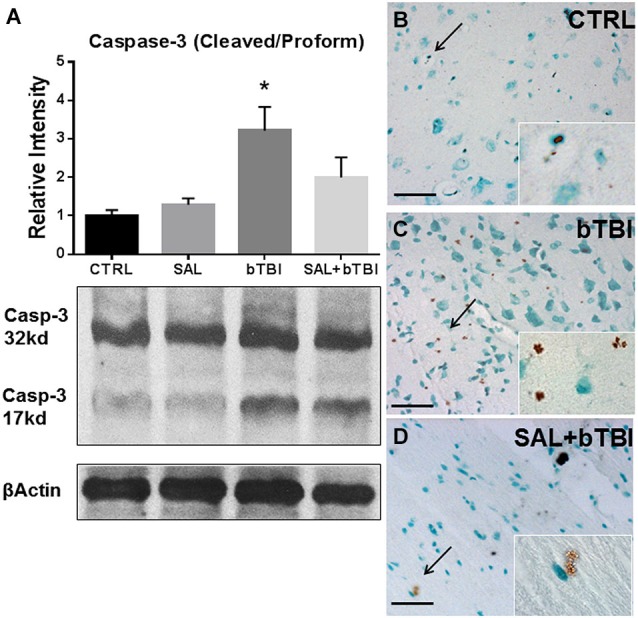
**Salubrinal reduces apoptosis in the left PFC following blast exposure. (A)** Caspase-3 cleavage increased in the left PFC of bTBI rats at 24 h (*p < 0.05 vs. SAL), but was not increased in SAL+bTBI rats at 24 h (*p* > 0.05 vs. SAL) (values represent mean ± s.e.m.; normalized to β-actin) (*n* = 5). **(B,C)** Evidence of increased cell membrane blebbing, an indicator of early apoptosis, was observed at 24 h in the left PFC of bTBI rats compared to control rats. **(D)** Membrane blebbing was reduced in the left PFC of SAL+bTBI rats. Images displayed at 20x; arrows demarcate insets at 63x. (Scale bars = 30 μm) (*n* = 4).

We wanted to determine if SAL had an effect on Caspase-3 cleavage. Caspase-3 cleavage is one of the final steps of the apoptotic cascade and is a common indicator used to assess cell death after TBI (Clark et al., 2000). One-Way ANOVA revealed a significant difference in Caspase-3 cleavage at 24 h post-bTBI (*F*_(3,16)_ = 5.533, *p* < 0.01). Caspase-3 cleavage significantly increased in the left PFC of bTBI rats (*t* = 3.251, *p* < 0.05), but was not significantly increased in the left PFC of SAL+bTBI rats (*t* = 1.200, *p* > 0.05; Bonferroni’s *post hoc* analysis; Figure 8A). These results suggest blast exposure elicits cell death through apoptosis. The results also suggest that ER stress plays an important role in the control of cellular fate following blast injury.

We wanted to determine if our blast model produced cell death, and if SAL pre-administration would reduce bTBI-induced cell death. To do this we employed a terminal deoxynucleotidyl transferase-mediated dUTP nick end labeling assay that utilizes DAB to stain cells undergoing the initial stages of apoptosis. We noticed cell membrane blebbing following blast exposure in the left PFC, which indicates early signs of apoptosis (Clark et al., 1997). Diaminobenzidine staining revealed increased apoptosis in bTBI rats compared to control rats (Figures 8B,C). Interestingly, SAL+bTBI rats displayed less DAB positive staining compared to bTBI rats (Figure 8D). The ratio of positive stained cells for control = 3/100, bTBI = 26/100, and SAL+bTBI = 3/100. *χ*^2^ = 33.456 with 2 degrees of freedom and *p* < 0.001.

### Impulsive-like behavior symptomatic of PFC damage is ameliorated by SAL

**Figure 9:**
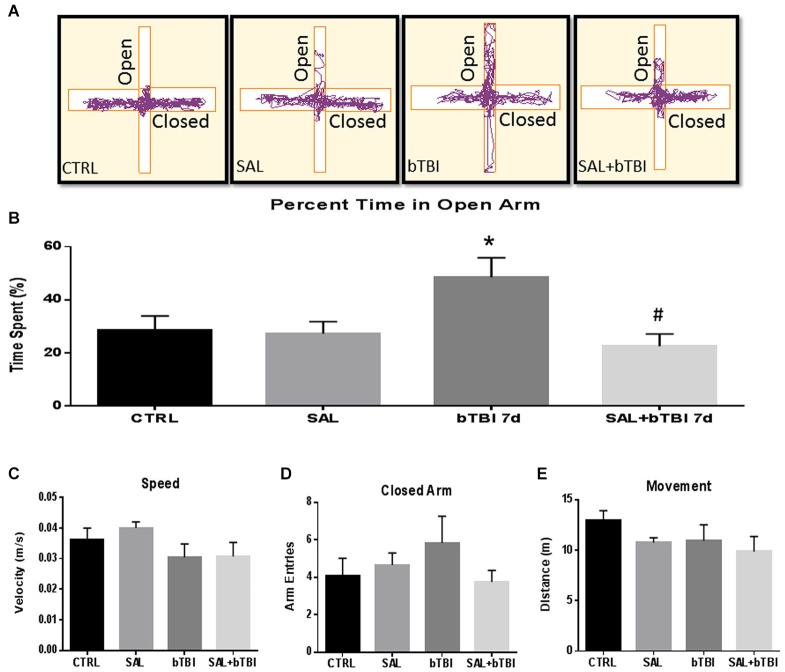
**Salubrinal ameliorates impulsive-like behavior indicative of PFC damage after Blast. (A)** A motion capture track plot of one animal from each experimental group during a single elevated plus maze (EPM) trial using AnyMaze Software™. **(B)** Percentage of time spent in the open arms of the EPM was significantly increased in bTBI rats compared to SAL only rats at 7 d (*p < 0.05 vs. SAL). Salubrinal administration prior to bTBI decreased the percent time spent in the open arms of the EPM (#*p* < 0.05 vs. bTBI) (values represent mean ± s.e.m.) (*n* = 12). No significant differences were observed between groups for **(C)** Speed, **(D)** Closed-Arm Entries, or **(E)** Movement within the EPM trials (5 min).

We assessed the percent time spent in the open arms of the EPM to measure changes in impulsive-like behavior (Mosienko et al., 2012). Track plots from the Anymaze™ analysis were included for each experimental group to visually support the behavioral changes (Figure 9A). A One-Way ANOVA exhibited a significant difference in the percent time spent in the open arms at 7 d post-bTBI (*F*_(3,44)_ = 4.250, *p* < 0.05). Tukey’s *post hoc* analysis revealed a significant increase in the percent time spent in the open arms between bTBI rats and SAL alone rats (*q* = 3.816, *p* < 0.05). SAL given prior to bTBI significantly reduced the percentage of time spent in the open arms compared to the 7 d post-bTBI group (*q* = 4.657, *p* < 0.05; Tukey’s *post hoc* analysis; Figure 9B). No significant differences were observed between groups in speed (*p* > 0.05; Figure 9C), closed arm entries (*p* > 0.05; Figure 9D), or total movement (*p* > 0.05; Figure 9E; Tukey’s *post hoc* analysis). Overall, these results indicate blast exposure may increase impulsive-like behavior, and that ER stress modulation may play a role in the manipulation of this type of behavior.

## Discussion

Data from the current study provides evidence that blast exposure disrupts the BBB and increases PERK-mediated ER stress in the left PFC. Impulsive-like behavior, a neuropsychiatric symptom associated with PFC damage (Johnson et al., 2013), was demonstrated by rats exposed to bTBI through their increased exploration in the open arms of the EPM. Immunohistochemistry results confirm bTBI increases the expression of neuronal apoptosis. Endoplasmic reticulum stress modulation, influences cellular fate and ameliorates impulsive-like behavior indicative of blast exposure. Overall, these results suggest a possible mechanistic link between ER stress, apoptosis and neuropsychiatric disease.

Previous groups have shown that disruptions in the BBB by various insults such as ischemic stroke and epilepsy can cause increased micro-vessel permeability (Kaya and Ahishali, 2011). The external forces of TBI can cause rapid depolarization of neuronal cell membranes; thereby, activating voltage gated Ca^2+^ channels and increasing intracellular Ca^2+^ levels (Gurkoff et al., 2013; Begum et al., 2014). Until recently, it was unknown to what extent blast injury disrupts the BBB. A mild exposure (~15 psi on incident recordings) from our clinically-relevant blast model previously exhibited no signs of brain hemorrhage under gross examination (Turner et al., 2013). A new study showed BBB disruption at 6 h following a mild intensity blast (~17.8 psi) in Sprague-Dawley rats (Abdul-Muneer et al., 2013). Our results indicate a more acute disruption in BBB permeability shown at 0.5 h, which is an earlier documentation of BBB disruption following bTBI. The rapid increase in permeability from blast injury is thought to result from an intracranial pressure spike (Chen et al., 2013b), and may be an important primary effect driving cellular stress (Arun et al., 2013).

Increased intracellular Ca^2+^ triggered ER stress and activated the UPR in other models of neuronal injury (Osada et al., 2009). Similarly, mild neurotrauma was shown to activate other adaptive arms of the UPR in mice (Rubovitch et al., 2011). The UPR is unique in that depending on the time and duration of the response, different arms of the pathway are activated (Rubovitch et al., 2011). If the UPR lasts too long, a switch from neuroprotection to apoptosis occurs (Nakagawa et al., 2000; Urra et al., 2013). Apoptosis is not purely detrimental to the damaged brain considering the heightened energy demands following neurotrauma. By limiting energy expended on severely damaged cells, the brain can preserve function to surviving cells.

Blast-induced CHOP elevation, along with increased Caspase-12 and Caspase-3 cleavage, suggests a neuronal shift from the repair response to apoptosis. Modulation of the ER stress response with SAL has been shown to attenuate CHOP expression (Zhang et al., 2014) and limit apoptosis in other models of neuronal injury (Sokka et al., 2007; Nakka et al., 2010). SAL’s effects on Caspase-12 remain controversial where some studies claim SAL mitigates Caspase-12 cleavage (Liu et al., 2012), while other studies claim SAL promotes cleavage (Gao et al., 2013). Furthermore, it is important to note that Caspase-12 is regulated through a calpain-dependent process (Nakagawa and Yuan, 2000) independent from the PERK-mediated UPR (Badiola et al., 2011). In our bTBI model, we show that SAL mitigates CHOP expression and reduces Caspase-3-mediated cell death with no effect on Caspase-12 cleavage. This suggests that SAL may not have a direct effect on Caspase-12 cleavage, but still plays a role in the control of cellular fate. Our findings, along with the findings of others, provide evidence for new ways to examine cellular stress and apoptosis in models of neuronal injury.

Neurotrauma is also intimately associated with post-injury changes in behavior (Schroeter et al., 2007). Blast exposure increases impulsive-like behavior in adult male Sprague-Dawley rats as shown in the EPM data. This finding may correlate with acute behavioral findings seen in soldiers following head injury (Menon et al., 2010). Interestingly, SAL attenuated the percentage of time spent in the open arms of the EPM following bTBI. These findings suggest that SAL may have a modulatory effect on impulsive-like behavior indicative of PFC damage through modulation of ER stress. While significant advances have been made in understanding the acute pathophysiology of blast exposure, it remains unclear how bTBI leads to the development of neuropsychiatric disorders (Tweedie et al., 2013).

Emerging evidence indicates that the UPR may be one potential mechanism linking acute neuronal injury and chronic disease pathology (Scheper and Hoozemans, 2013). In various models of neurodegenerative disease, evidence of PERK-mediated ER stress activation has been implicated (Costa et al., 2012; Ho et al., 2012; Nijholt et al., 2012). Moreover, experimental work using other models of neurotrauma have shown elevated UPR markers (Farook et al., 2013; Begum et al., 2014) and behavioral deficits (Goldstein et al., 2012; Petraglia et al., 2014). Based on our findings, the elevation of UPR markers concurrently with Caspase-3 cleavage suggests neuronal apoptosis, which has been implicated as an early indicator of chronic disease pathology (McKee et al., 2009). As such, our study provides a correlative link between blast-induced UPR activation and neuropsychiatric disorder development. Future studies, likely using genetically altered animals, or additional pharmacologic inhibitors, are required to examine the precise role of ER stress in the development of chronic disease following neurotrauma. Similarly, investigating PERK-mediated ER stress in pathologic clinical specimens will further clarify the potential role of the pathway in neuropsychiatric disease development.

Long-term studies utilizing both a single and repetitive injury model are warranted in order to examine not only chronic disease processes associated with ER stress but also the effect of repetitive injury on the activation of the ER stress response. Finally, the contribution of the other two adaptive arms of the UPR following blast injury remains to be elucidated. All three adaptive arms of the UPR share CHOP as a signal mediator and is of particular interest due to the overlapping nature of the pathways and the potential for modulation of signaling in a biphasic manner. We are also interested in the role of axonal shearing as a result of blast exposure. We have shown previously that a majority of damage from our blast model occurs in the corpus callosum (Turner et al., 2013). This is considered another primary effect of blast exposure and warrants future investigation using our model.

Blast exposure disrupts the BBB and triggers ER stress in the left PFC. Rats exposed to blast exhibit more impulsive-like behavior and display markers of neuronal apoptosis. When bTBI rats were given the ER stress modulator, SAL, markers of apoptosis and impulsive-like behavior were both attenuated. The cells that survive the initial primary injury of bTBI are those that we seek to protect from secondary injury mechanisms. Future studies linking ER stress to chronic disease are ongoing and could provide new molecular targets for treatment following blast injury.

## Conflict of interest statement

The authors declare that the research was conducted in the absence of any commercial or financial relationships that could be construed as a potential conflict of interest.
